# Single versus double transseptal puncture for atrial fibrillation radiofrequency ablation: a systematic review and meta-analysis

**DOI:** 10.3389/fcvm.2026.1907029

**Published:** 2026-10-05

**Authors:** Jie Xu, Wenqiang Zhang, Feilin Ye, Junning Lei, Kun Chen, Hui Li

**Affiliations:** 1 Department of Cardiology, 986th Hospital, Xijing Hospital, Fourth Military Medical University, Xi’an, China; 2 Department of Anatomy, Histology and Embryology and K.K. Leung Brain Research Center, Fourth Military Medical University, Xi'an, China

**Keywords:** atrial fibrillation, meta-analysis, radiofrequency ablation, single, transseptal puncture

## Abstract

**Background:**

The single puncture technique has emerged as an alternative to conventional double transseptal puncture for radiofrequency ablation of atrial fibrillation (AF). This meta-analysis aimed to compare the efficacy and safety of these two approaches.

**Methods:**

We systematically searched PubMed, Embase, and the Cochrane Library up to 1st March, 2026. Prospective and retrospective cohort studies comparing single vs. double transseptal puncture during AF ablation were included. The primary outcome was the sinus rhythm maintenance. Secondary outcomes included total procedure time, fluoroscopy time, and periprocedural complications. Data were pooled using a random-effects model, and heterogeneity was assessed with the I² statistic.

**Results:**

Four studies involving 1811 patients were included. No significant difference was observed in 3-month sinus rhythm maintenance between the single- and double-puncture groups (OR 1.20, 95% CI: 0.95–1.51, *P* = 0.13). While fluoroscopy time presented similar values (MD −0.84, 95% CI: −4.80–3.11, *P* = 0.68), procedure duration was significantly longer with single transseptal puncture (MD 11.65, 95% CI: 2.62–20.69, *P* = 0.01). The incidence of periprocedural complications was similar between the two strategies (OR 0.81, 95% CI: 0.47–1.38, *P* = 0.43).

**Conclusions:**

Single transseptal puncture represents an effective and safe alternative to double puncture for atrial fibrillation ablation, with comparable rhythm outcomes, fluoroscopy exposure and complication rates, despite longer procedural duration.

**Systematic Review Registration:**

https://www.crd.york.ac.uk/prospero/view/CRD420261300869, Identifier: CRD420261300869.

## Introduction

Transseptal puncture (TSP) is an essential step for left atrial access during radiofrequency ablation of atrial fibrillation (AF). Accurate and safe TSP is critical, as it directly influences both the feasibility and safety of the ablation procedure (1). The conventional approach for AF ablation typically involves a double TSP to accommodate both a circular mapping catheter and a separate ablation catheter. This dual puncture technique allows for simultaneous catheter positioning and, importantly, enables real time monitoring of pulmonary vein potentials throughout the ablation process without the need for catheter exchange (2).

An alternative strategy utilizes a single puncture site to advance multiple catheters into the left atrium through a single sheath. This simplified approach reduces procedural steps and may enhance workflow efficiency (3). Moreover, emerging evidence suggests that single TSP may be associated with a lower risk of complications by eliminating the need for a second septal crossing, potentially reducing the likelihood of pericardial effusion, air embolism, or thrombus formation (4).

However, while single TSP avoids a second puncture, it requires sequential catheter exchanges, which may prolong procedure time and precludes real time monitoring of pulmonary vein potentials during ablation, a limitation that could theoretically compromise long term freedom from AF recurrence (5). Given these competing considerations, we performed a systematic review and meta-analysis comparing single vs. double TSP for AF ablation, with particular focus on sinus rhythm maintenance, procedural efficacy, total procedure duration, fluoroscopy time, and complication rates.

## Methods

### Search strategy

The present systematic review and meta-analysis followed the PRISMA guidelines. Prior to initiation, the study protocol was registered with the International Prospective Register of Systematic Reviews (PROSPERO), identifier CRD420261300869. As this study involved no collection of individual patient data, formal ethical review was not required.

A comprehensive literature search was conducted in Medline, Embase, and the Cochrane Library databases up to 1st March, 2026 by two independent reviewers (Jie Xu and Junning Lei). The search strategy combined the terms “atrial fibrillation” or “pulmonary vein isolation” and “transseptal puncture” or “transseptal access” or “transseptal approach” or “atrial septal puncture” or “transeptal puncture” or “trans-septal” and “single” or “unilateral” or “once” or “solely” and “double” or “dual” or “twice” or “bilateral”. We placed no restrictions on language, publication date, or status. Eligible study designs included clinical trials and observational studies. Reference lists of included articles were also manually screened for additional eligible studies.

### Study design

Studies were eligible if they met the following criteria: (1) clinical trial; (2) inclusion of patients with persistent or paroxysmal AF undergoing first-time ablation; (3) comparison of radiofrequency ablation with single TSP vs. double TSP; and (4) reported atrial tachyarrhythmia recurrence with minimum 3-month follow-up. We excluded studies involving redo procedures, and those published only as abstracts or conference presentations. Two investigators (Wenqiang Zhang and Feilin Ye) independently screened all records, with disagreements resolved by consensus or consultation with a third author (Kun Chen).

### Data extraction

From each included study, two independent researchers (Jie Xu and Junning Lei) extracted the data: (1) study characteristics: first author, publication year, inclusion period, and ablation strategy; (2) patient characteristics: sample size per group, age, sex, CHA₂DS₂-VASc score, left atrial diameter, and left ventricular ejection fraction; and (3) outcomes: primary endpoint definition (sinus rhythm maintenance), follow-up duration, procedure time, fluoroscopy time, and complications. Data were collected when available for cohort characterization. When incomplete or missing outcome data were encountered in original publications, we only extracted analyzable data as reported. We did not contact authors of primary studies to obtain additional raw data, and no statistical imputation was performed for missing values.

### Quality assessment

Two reviewers (Wenqiang Zhang and Feilin Ye) independently evaluated the methodological quality of each included study. the Cochrane Risk of Bias (RoB) tool was predefined for assessing the quality of randomized controlled trials (RCTs), while the Newcastle-Ottawa Quality Assessment Scale (NOS) was applied for observational studies. After comprehensive literature screening, no eligible RCTs were finally included. Therefore, the methodological quality of all included observational studies was assessed using the NOS scale. In this scale, three domains with a total of nine points were involved. Each domain was rated, and stars were awarded for each quality item, with a maximum of nine stars overall. Disagreements were resolved through consultation with a third author (Kun Chen). A summary of the quality assessment for each study is provided in Supplementary Table S1.

### Outcomes

The primary endpoint was freedom from any sustained atrial arrhythmia (including AF, atrial tachycardia, or atrial flutter) off antiarrhythmic drugs following a 3-month blanking period after the ablation procedure.

Secondary endpoints included: (i) total procedure duration; (ii) total fluoroscopy time; and (iii) procedural complications. Safety outcomes included overall procedural complications.

### Statistical analysis

All meta-analyses were performed using a random-effects model. Continuous variables are reported as mean ± standard deviation, while categorical variables are presented as frequencies and percentages. Pooled odds ratios (OR) and weighted mean differences (MD) with 95% confidence intervals (CI) were calculated for each outcome. Statistical significance was defined as *p* < 0.05. Between-study heterogeneity was assessed using the *I²* statistic, with thresholds defined as: no (0%), low (<25%), moderate (25%–50%), and high (>50%). Sensitivity analysis was conducted by sequentially omitting one study at a time to evaluate the influence of individual studies on the overall effect estimates. All analyses were performed using Review Manager 5.4 and SPSS version 29.

## Results

### Study selection and quality assessment

A total of 183 records were identified. After removing duplicates and excluding studies that did not meet the inclusion criteria based on title and abstract screening, 17 studies remained for full-text assessment. Following full-text review, 13 studies were excluded for the following reasons: 4 did not report the primary outcome, 6 lacked a control group, 2 were only available as abstracts and 1 included repeated participants. As a result, four studies were included in the final analysis: Stauber 2021 (6), Yu 2019 (7), Silva 2023 (8), Park 2021 (9). The study selection process is illustrated in Figure 1. Overall, all included studies were assessed as having a low risk of bias (NOS score≥8, Supplementary Table S1).

**Figure 1:**
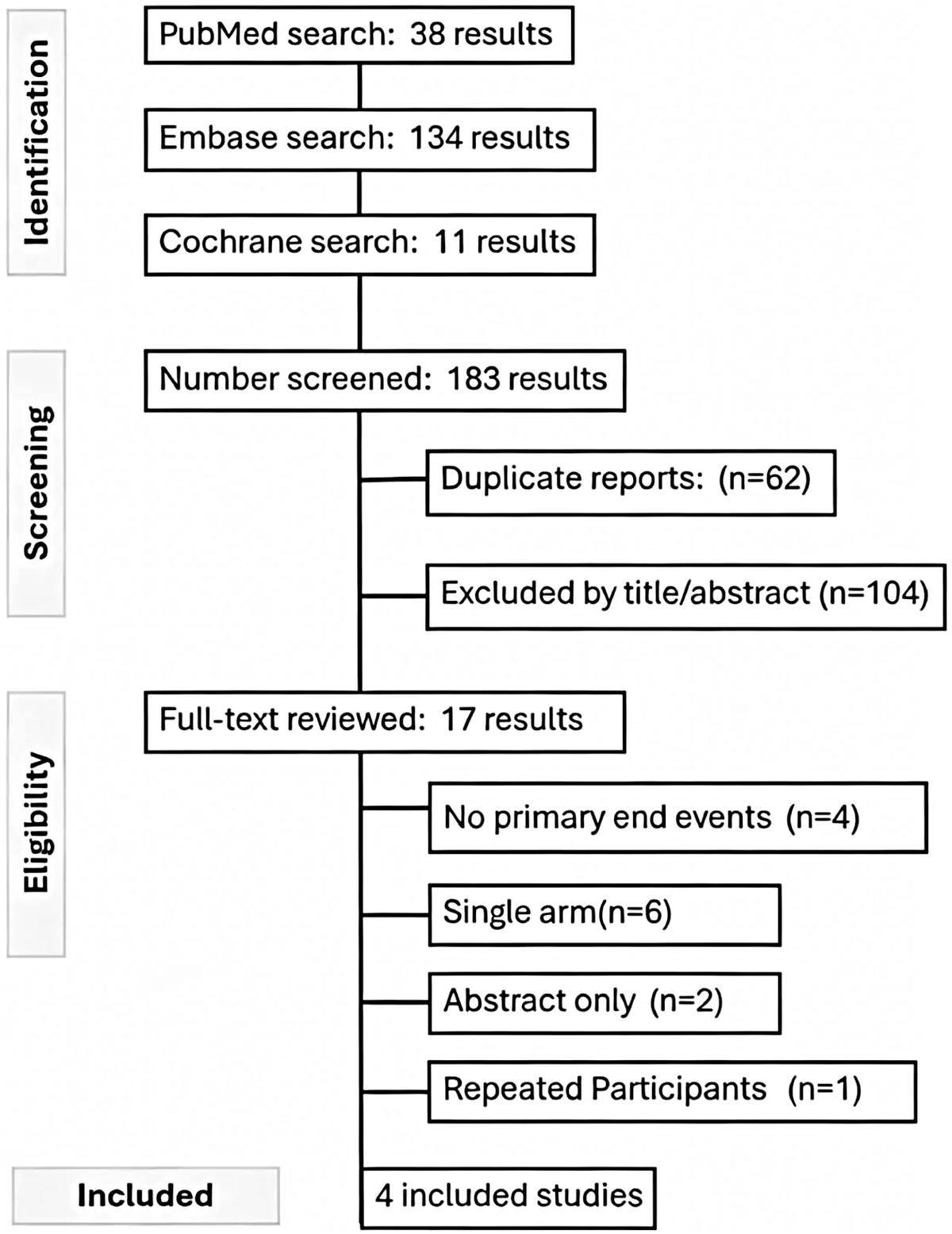
Study selection flowchart.

### Study characteristics

The design and characteristics of the studies included in the analysis are detailed in Table 1. A total of 1,811 patients with persistent or paroxysmal AF were included, of whom 878 patients underwent single TSP while 933 patients underwent double TSP. The selected studies were published between 2019 and 2023. In only one study, the patients underwent PVI alone, whereas the other three included CTI or other additional ablation lines. Study designs included prospective and retrospective cohorts. The baseline characteristics are listed in Table 2. The two groups were well balanced with respect to age, left ventricular ejection fraction, and left atrial diameter, and no statistically significant differences were observed.

**Table 1:** Summary of study characteristics.

Study	Year	Country	Study design	AF subtype	Intervention	Control	Follow-up, months	Ablation strategy
Stauber	2021	Germany	Prospective	32.0% Paroxysmal or 68.0% persistent AF	Single TSP	Double TSP	6	CPVI, Linear ablations if necessary
Yu	2019	Korea	Prospective	Paroxysmal AF	Single TSP	Double TSP	20.2 ± 8.7	CPVI + CTI
Silva	2023	Portugal	Retrospective	67.7% Paroxysmal or 32.3% persistent AF	Single TSP	Double TSP	24	CPVI
Park	2021	Korea	Prospective	75.0% Paroxysmal or 25.0% persistent AF	Single TSP	Double TSP	17.8 ± 13.8	CPVI + CTI

AF: atrial fibrillation; CPVI, circumferential pulmonary vein isolation; CTI, cavo tricuspid Isthmus; TSP, transseptal puncture.

**Table 2:** Baseline of population characteristics.

Study	Patients, N	Age, years	Male, N(%)	CHA_2_DS_2_-VASC score	Inclusion date	LVEF, %	LAD, mm
Stauber	ST = 202; DT = 276	62.7 ± 11.9	310 (64.9%)	2.0 ± 1.5	01/2014 to 09/2014	58.2 ± 9.7	44.0 ± 5.9
Yu	ST = 148; DT = 74	59.4 ± 10.7	154 (69.4%)	1.8 ± 1.6	04/2015 to 06/2017	64.7 ± 7.3	39.5 ± 5.7
Silva	ST = 115; DT = 170	59.5 ± 11.6	181 (63.5%)	1.7 ± 1.3	07/2015 to 03/2020	LVEF < 35%, 11 (4.1%) LVEF < 50%, 36 (13.4%) LVEF ≥ 50%, 238 (82.5%)	44.0 ± 8.5
Park	ST = 413; DT = 413	59.5 ± 10.7	587 (71.1%)	1.6 ± 1.4	10/2015 to 01/2020	64.2 ± 8.1	49.6 ± 4.4

ST, single transseptal puncture; DT, double transseptal puncture; LVEF, left ventricular ejection fractions; LAD, left atrial diameter.

### Freedom from atrial arrhythmia

All studies reported freedom from any sustained atrial arrhythmia, after a blanking period of 3 months and 6–24 months follow-up. A total of 1,811 patients (878 in the Single TSP group and 933 in the Double TSP group) were evaluated for the endpoint. 719 (81.9%) in the Single TSP group and 725 (77.7%) in the Double TSP group were reported freedom from atrial arrhythmia. Pooled analysis revealed no statistically significant difference in overall event rates between the Single TSP and Double TSP strategies (OR 1.20, 95% CI 0.95–1.51, *P* = 0.13, I² = 0%) (Figure 2).

**Figure 2:**
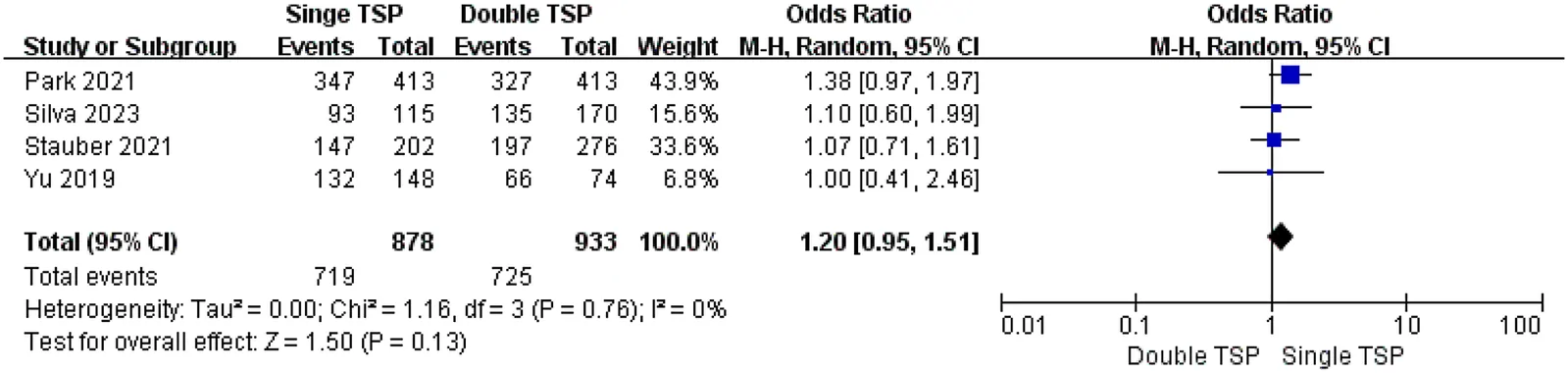
Pooled analysis for freedom from any atrial arrhythmia comparing single TSP and double TSP. Numbers displayed represent OR with 95% CI. CI: confidence intervals; OR: odds risk; TSP: Transseptal puncture. Single TSP is represented “right” and Double TSP represented “left”.

### Total procedure duration

As expected, the procedure duration was significantly longer in the Single TSP group (MD 11.65, 95% CI: 2.62–20.69, *P* = 0.01). However, substantial heterogeneity was observed across studies (I² = 83%, *P* < 0.001) (Figure 3). To explore the source of this heterogeneity, a sensitivity analysis was conducted by omitting one study at a time. Notably, exclusion of the study by Park et al. (2021) resulted in a dramatic reduction in heterogeneity (I² dropped to 0%, *P* = 0.61), while the overall conclusion remained unchanged; the Single TSP procedure was still associated with a significantly longer operative time (MD 7.05, 95% CI: 2.66–11.44, *P* = 0.002) (Supplementary Figure S2).

**Figure 3:**
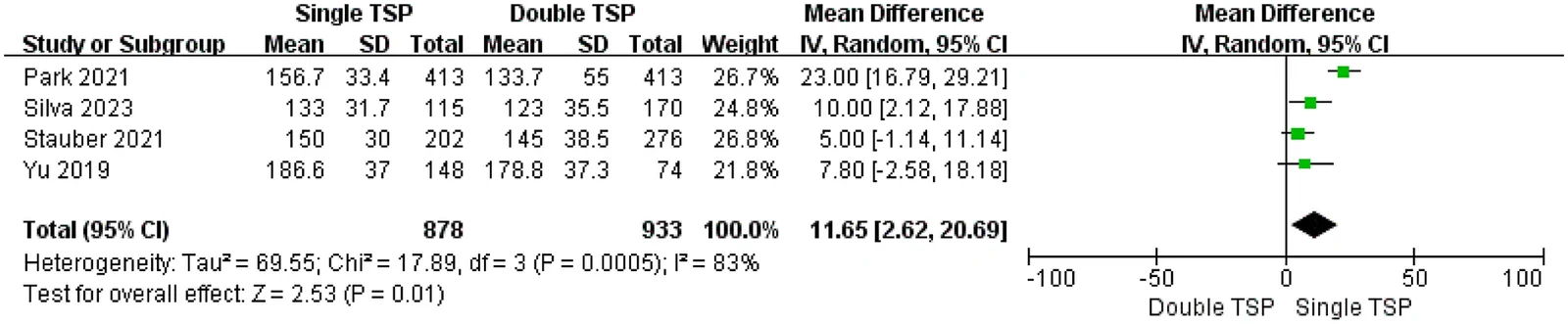
Pooled analysis for procedure duration comparing single TSP and double TSP. Data displayed Mean Difference with 95% CI. CI: confidence intervals; TSP, Transseptal puncture. Single TSP is represented “right” and Double TSP represented “left”.

### Fluoroscopy time during the procedure

Across four studies comparing fluoroscopy time between the Single TSP and Double TSP groups, the random effects pooled analysis showed no significant overall difference (MD −0.84, 95% CI: −4.80–3.11, *P* = 0.68), indicating comparable fluoroscopy exposure between the two strategies (Figure 4). The high degree of heterogeneity observed across studies is likely due to differences in center-specific protocols, operator experience and procedural complexity. Given the limited number of studies and the high degree of variability across trials, no further subgroup or sensitivity analyses were performed.

**Figure 4:**
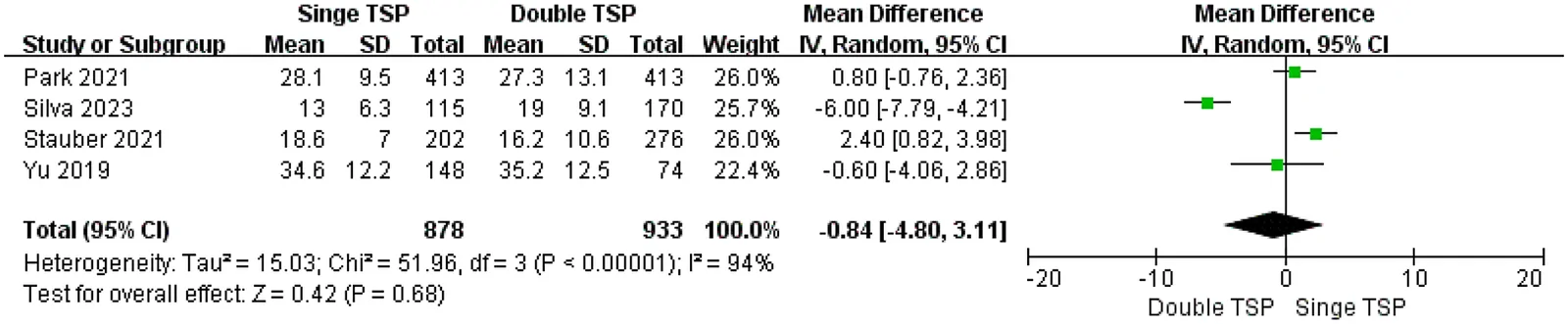
Pooled analysis for fluoroscopy time comparing single TSP and double TSP. Data displayed Mean Difference with 95% CI. CI: confidence intervals; TSP, Transseptal puncture. Single TSP is represented “right” and Double TSP represented “left”.

### Periprocedural complications

Procedural complications occurred in 46 of 878 patients (5.2%) in the Single TSP group and in 59 of 933 patients (6.3%) in the Double TSP group. Pooled results showed no statistically significant difference between the Single TSP and Double TSP groups (OR 0.81, 95% CI: 0.47–1.38, *P* = 0.43). The heterogeneity test indicated moderate inter-study heterogeneity (I² = 34%, *P* = 0.21), which was deemed acceptable for pooled analysis. This indicates that both puncture techniques were associated with a comparable rate of periprocedural complications (Figure 5).

**Figure 5:**
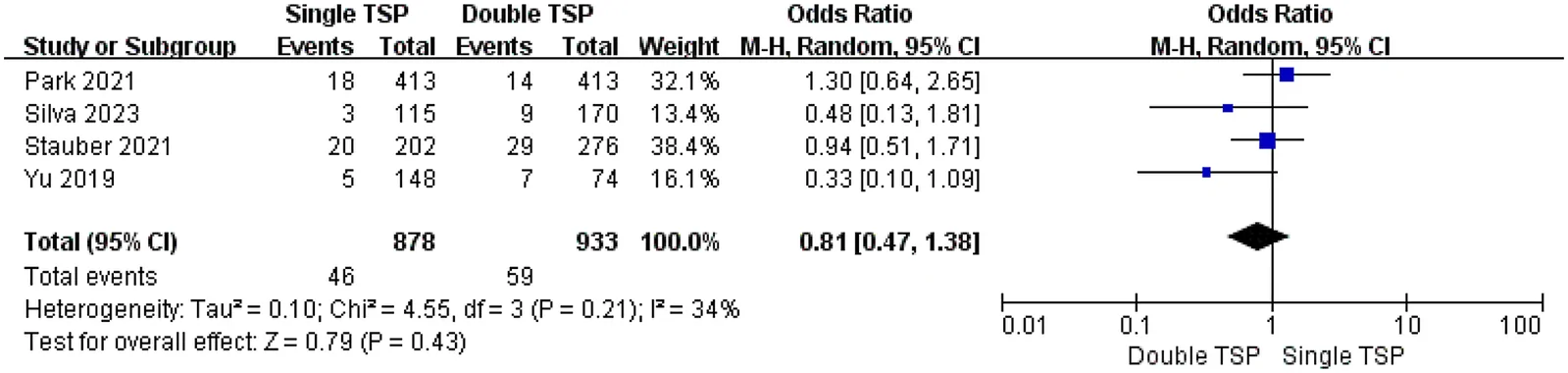
Pooled analysis for overall complications comparing single TSP and double TSP. Numbers displayed represent OR with 95% CI. CI, confidence intervals; OR, odds risk; TSP, Transseptal puncture. Single TSP is represented “right” and Double TSP represented “left”.

## Discussion

In this systematic review and meta-analysis of 4 studies comprising 1,811 patients with atrial fibrillation, we compared the efficacy and safety of single vs. double transseptal puncture for radiofrequency ablation. The rate of sustained atrial arrhythmia was similar between groups. There is no significant difference in fluoroscopy exposure or complication rates between single and double transseptal puncture techniques, although single puncture was associated with a longer total procedure duration. These findings suggest that a single puncture strategy still represents a feasible alternative for atrial fibrillation ablation.

### Real time monitoring and freedom from AT

The traditional double transseptal puncture approach in atrial fibrillation radiofrequency ablation enables the placement of separate mapping and ablation catheters in the left atrium. This configuration allows for real time monitoring of pulmonary vein potential (PVP) elimination during energy delivery, a technical feature previously associated with confirmed procedural efficacy and durable pulmonary vein isolation (10). In contrast, the single transseptal puncture strategy, which relies on a single catheter for sequential mapping and ablation, precludes real time PVP visualization during ablation; confirmation of electrical isolation is therefore deferred until post-ablation reassessment. Even though, advances in three-dimensional mapping systems have partially addressed this limitation by improving the accuracy of lesion assessment and ensuring durable ablation despite the lack of real time PVP feedback (11). Despite this theoretical limitation in intraprocedural verification, early clinical observations suggested comparable acute outcomes (12). This was substantiated by a large retrospective study in 2025, which reported no significant difference in the rate of sinus rhythm maintenance during the index hospitalization (13). The present meta-analysis extends these findings by demonstrating that long term efficacy, defined as freedom from sustained atrial arrhythmia after the 3-month blanking period, was also comparable between the single and double puncture groups. The cumulative data indicates that the lack of real time PVP monitoring with a single puncture strategy does not compromise long term clinical outcomes.

### Time and radiation efficiency

The unadjusted pooled meta-analysis data demonstrated prolonged total procedural duration in the single TSP group. However, such time discrepancy cannot be solely attributed to the transseptal puncture strategy, due to the confounding effects of various procedures, additional ablations (e.g., CTI ablations), left atrial dwelling time and learning curve of the operators. Traditionally, this temporal trade-off was deemed acceptable when weighed against the expected reduction in radiation exposure, a benefit with well documented implications for the health of patients and staff (14). However, the present meta-analysis did not demonstrate a significant reduction in fluoroscopy time with the single puncture approach. This finding may be explained by the fact that the included studies predominantly reflect practices where operators still rely on fluoroscopic guidance as a safety backup, even when using three-dimensional electroanatomical mapping (T3D) and intracardiac echocardiography (ICE) (15).

In recent years, several centers have reported the feasibility of performing transseptal puncture without any ionizing radiation (16, 17). Of note, these zero-fluoroscopy techniques are predominantly employed with a single puncture approach (18), which may drastically reduce radiation exposure compared to conventional methods. Importantly, emerging evidence suggests that in the context of zero or ultra-low fluoroscopy single puncture procedures, the total procedure duration is not significantly prolonged compared to conventional approaches, while the reduction in radiation exposure remains substantial (19, 20). Notably, the advent of novel technologies such as pulsed field ablation (PFA), which typically mandates a single puncture for the delivery of its larger diameter dedicated sheaths, further underscores the importance of optimizing single access techniques (21).

Nevertheless, the abstract data from the “Go for Zero Fluoroscopy registry”, which included both single and double puncture strategies, offer a contrasting perspective: the single TSP approach was associated with significantly higher dose-area-product, longer fluoroscopy time, and longer overall procedure time compared to the double puncture strategy (22). This technological evolution provides a new platform for extending the conclusions of the present study and highlights the need for further research to reconcile these conflicting observations, particularly in the era of zero-fluoroscopy and novel ablation modalities.

### Periprocedural complications

From a theoretical perspective, single transseptal puncture may lower periprocedural complications by avoiding a second septal puncture and related manipulations. Nevertheless, this meta-analysis found no significant intergroup difference in complication rates, which can be explained by the inherently low complication incidence of atrial fibrillation ablation and the limited sample size of included studies, restricting the ability to identify subtle differences in rare adverse events.

Velasco et al. study (2025), encompassing over 200,000 patients, demonstrated that the single transseptal puncture approach was associated with a comparable but numerically lower risk of total procedural complications, with the larger cohort providing greater precision to characterize the safety profile (13). Specifically, the reduction in complications appeared driven by lower rates of pericardial effusion and access-site adverse events, both of which are plausibly linked to the reduced number of transseptal manipulations and vascular instrumentation. Nevertheless, existing data also indicate a small elevated risk of air embolism among patients receiving single TSP. Importantly, this incremental risk did not translate into statistically significant differences in clinical outcomes. Collectively, these findings suggest that while individual studies may be underpowered to detect differences in complication rates, the aggregate evidence from large scale real world data favors the safety of the single puncture strategy. The consistency of this directional signal across different study designs reinforces the clinical relevance of this technical modification.

Advancements in transseptal access techniques have introduced several innovative tools, including visualized steerable sheath (23), intracardiac ultrasound, electrocautery-assisted puncture, and dedicated transseptal guidewires, together with zero-fluoroscopy procedural strategies (1). These innovations are reshaping clinical practice and gradually changing the application scenarios for conventional single- and double-transseptal puncture techniques. Nevertheless, traditional transseptal puncture approaches remain clinically meaningful for the majority of routine electrophysiology centers, where these advanced tools may not be universally available. Therefore, findings from the present meta-analysis offer practical real-world evidence for centers performing conventional radiofrequency ablation. Future high-quality comparative studies are warranted to further evaluate single vs. double TSP when combined with these modern puncture-related technologies.

### Limitation

This meta-analysis has several limitations. First, the observational or prospective nature of included studies introduces potential selection bias and confounding, as the choice of puncture strategy may depend on patient anatomy and procedural complexity. Moreover, observed time discrepancies cannot be solely attributed to puncture approaches, given confounding from variable ablation protocols, additional CTI ablation, left atrial dwelling time and operator learning-curve effects. Second, significant heterogeneity in procedural outcomes limits the generalizability of our findings. Third, despite our comprehensive literature search, the small number of included studies precluded the use of statistical tests (e.g., Egger’s test) to robustly assess publication bias, meaning that potential bias cannot be ruled out. These limitations warrant caution in interpretation, and large scale prospective randomized trials are needed to confirm these findings.

## Conclusion

In conclusion, for atrial fibrillation radiofrequency ablation, single transseptal puncture serves as an effective and safe alternative to double transseptal puncture, as the two strategies yield comparable rhythm outcomes, fluoroscopy exposure and complication rates, despite the longer procedural duration associated with the single puncture approach.

## Data Availability

The original contributions presented in the study are included in the article/Supplementary Material, further inquiries can be directed to the corresponding author/s.

## References

[1] Kaplan RM Wasserlauf J Knight BP. Transseptal access: a review of contemporary tools. J Cardiovasc Electrophysiol. (2022) 33(8):1927–31. 10.1111/jce.15428 35229417 PMC9545840

[2] Haegeli LM Wolber T Ercin E Altwegg L Krasniqi N Novak PG. Double transseptal puncture for catheter ablation of atrial fibrillation: safety of the technique and its use in the outpatient setting. Cardiol Res Pract. (2010) 2010:295297. 10.4061/2010/295297 21197071 PMC3004379

[3] Akdis D Weidmann L Guan F Bachmann M Winnik S Duru F. Clinical experience of pulmonary vein isolation via single transseptal puncture in atrial fibrillation patients: comprehensive characterization and follow-up. Int J Cardiol. (2025) 418:132557. 10.1016/j.ijcard.2024.132557 39276818

[4] Fagundes R Mantica M De Luca L Forleo G Pappalardo A Avella A. Safety of single transseptal puncture for ablation of atrial fibrillation: retrospective study from a large cohort of patients. J Cardiovasc Electrophysiol. (2007) 18(12):1277–81. 10.1111/j.1540-8167.2007.00958.x 17883403

[5] Efremidis M Letsas K Giannopoulos G Lioni L Vlachos K Asvestas D. Early pulmonary vein reconnection as a predictor of left atrial ablation outcomes for paroxysmal atrial fibrillation. Europace. (2015) 17(5):741–6. 10.1093/europace/euu216 25687747

[6] Stauber A Kornej J Sepehri Shamloo A Dinov B Bacevicius J Dagres N. Impact of single versus double transseptal puncture on outcome and complications in pulmonary vein isolation procedures. Cardiol J. (2021) 28(5):671–7. 10.5603/CJ.a2020.0037 32207839 PMC8428934

[7] Yu HT Shin DG Shim J Nam G-B Yoo WW Lee JH. Unilateral versus bilateral groin puncture for atrial fibrillation ablation: multi-center prospective randomized study. Yonsei Med J. (2019) 60(4):360–7. 10.3349/ymj.2019.60.4.360 30900422 PMC6433563

[8] Silva Cunha P Teixeira BL Laranjo S Portugal G Valente B Delgado AS. A simplified single transseptal puncture approach using high-density 3D voltage mapping for atrial fibrillation ablation: acute complications and long-term results. Front Cardiovasc Med. (2023) 10:1309900. 10.3389/fcvm.2023.1309900 38075955 PMC10701513

[9] Park YJ Yu HT Kim T-H Uhm J-S Joung B Lee M-H. Effectiveness and safety of single transseptal ablation for atrial fibrillation in real-word practice. Clin Cardiol. (2021) 44(4):547–54. 10.1002/clc.23574 33615493 PMC8027586

[10] Sauer WH McKernan ML Lin D Gerstenfeld EP Callans DJ Marchlinski FE. Clinical predictors and outcomes associated with acute return of pulmonary vein conduction during pulmonary vein isolation for treatment of atrial fibrillation. Heart Rhythm. (2006) 3(9):1024–8. 10.1016/j.hrthm.2006.05.007 16945795

[11] Boersma LV Van Der Voort P Debruyne P Dekker L Simmers T Rossenbacker T. Multielectrode pulmonary vein isolation versus single tip wide area catheter ablation for paroxysmal atrial fibrillation: a multinational multicenter randomized clinical trial. Circ Arrhythm Electrophysiol. (2016) 9(4):e003151. 10.1161/CIRCEP.115.003151 27071830

[12] Reddy VY Morales G Ahmed H Neuzil P Dukkipati S Kim S. Catheter ablation of atrial fibrillation without the use of fluoroscopy. Heart Rhythm. (2010) 7(11):1644–53. 10.1016/j.hrthm.2010.07.011 20637313

[13] Velasco A Diaz Delgado PJ Mellado S Rodriguez Lopez J Alviz I Dong H. Procedural outcomes of single versus double transeptal access for ablation of atrial fibrillation: data from the NCDR afib ablation registry. J Interv Card Electrophysiol. (2026) 69(4):617–25. 10.1007/s10840-025-02196-4 41428032 PMC13221313

[14] Tahin T Riba A Nemeth B Arvai F Lupkovics G Szeplaki G. Implementation of a zero fluoroscopic workflow using a simplified intracardiac echocardiography guided method for catheter ablation of atrial fibrillation, including repeat procedures. BMC Cardiovasc Disord. (2021) 21(1):407. 10.1186/s12872-021-02219-8 34433424 PMC8390247

[15] Pittet T Delacrétaz E Cook S Vivekanantham H. An approach to fluoroless radiofrequency atrial fibrillation ablation. Front Cardiovasc Med. (2025) 12:1524426. 10.3389/fcvm.2025.1524426 39911561 PMC11794273

[16] Debreceni D Janosi K Bocz B Turcsan M Lukacs R Simor T. Zero fluoroscopy catheter ablation for atrial fibrillation: a systematic review and meta-analysis. Front Cardiovasc Med. (2023) 10:1178783. 10.3389/fcvm.2023.1178783 37396578 PMC10313423

[17] Shawki M Ilyas RP Chauhan K Rodrigues TS Nabhani SL Wynn G. Zero-fluoroscopy ablation methods for atrial fibrillation: a systematic review and meta-analysis. Pacing Clin Electrophysiol. (2026). 10.1111/pace.70277 42099078

[18] Baykaner T Quadros KK Thosani A Yasmeh B Mitra R Liu E. Safety and efficacy of zero fluoroscopy transseptal puncture with different approaches. Pacing Clin Electrophysiol. (2020) 43(1):12–8. 10.1111/pace.13841 31736095 PMC7383937

[19] Haegeli LM Stutz L Mohsen M Wolber T Brunckhorst C On C-J. Feasibility of zero or near zero fluoroscopy during catheter ablation procedures. Cardiol J. (2019) 26(3):226–32. 10.5603/CJ.a2018.0029 29611170 PMC8086663

[20] Wang Y Long D Xiao F Chen M Liu X Zhang J. Multicenter practice of non/minimized fluoroscopy ablation for paroxysmal AF in China: the PAF-ICE trial. JACC Asia. (2025) 5(12):1540–8. 10.1016/j.jacasi.2025.07.011 40910962 PMC12794002

[21] Tohoku S Bordignon S Schaack D Hirokami J Urbanek L Urbani A. Initial real-world data on catheter ablation in patients with persistent atrial fibrillation using the novel lattice-tip focal pulsed-field ablation catheter. Europace. (2024) 26(6):euae129. 10.1093/europace/euae129 38916275 PMC11197047

[22] Miskowiec D Qawoq HD Zycinski P Kosiuk J Chudzik M Kasprzak JD. Single versus double transseptal puncture for pulmonary vein isolation with radiofrequency catheter ablation for atrial fibrillation—insights from “go for zero fluoroscopy” registry. EP Europace. (2024) 26(Supplement_1):euae102.081. 10.1093/europace/euae102.081

[23] Guo R Jia R Cen Z Lu S Yang C Han S. Effects of the visualized steerable sheath applied to catheter ablation of paroxysmal atrial fibrillation. J Interv Card Electrophysiol. (2022) 64(2):511–8. 10.1007/s10840-021-01096-7 34850310

